# Modulation of the Surface Proteome through Multiple Ubiquitylation Pathways in African Trypanosomes

**DOI:** 10.1371/journal.ppat.1005236

**Published:** 2015-10-22

**Authors:** Martin Zoltner, Ka Fai Leung, Sam Alsford, David Horn, Mark C. Field

**Affiliations:** 1 Division of Biological Chemistry and Drug Discovery, University of Dundee, Dundee, United Kingdom; 2 Department of Pathology, University of Cambridge, Cambridge, United Kingdom; 3 London School of Hygiene and Tropical Medicine, Keppel Street, London, United Kingdom; University of California, Los Angeles, UNITED STATES

## Abstract

Recently we identified multiple suramin-sensitivity genes with a genome wide screen in *Trypanosoma brucei* that includes the invariant surface glycoprotein ISG75, the adaptin-1 (AP-1) complex and two deubiquitylating enzymes (DUBs) orthologous to ScUbp15/HsHAUSP1 and pVHL-interacting DUB1 (type I), designated TbUsp7 and TbVdu1, respectively. Here we have examined the roles of these genes in trafficking of ISG75, which appears key to suramin uptake. We found that, while AP-1 does not influence ISG75 abundance, knockdown of TbUsp7 or TbVdu1 leads to reduced ISG75 abundance. Silencing TbVdu1 also reduced ISG65 abundance. TbVdu1 is a component of an evolutionarily conserved ubiquitylation switch and responsible for rapid receptor modulation, suggesting similar regulation of ISGs in *T*. *brucei*. Unexpectedly, TbUsp7 knockdown also blocked endocytosis. To integrate these observations we analysed the impact of TbUsp7 and TbVdu1 knockdown on the global proteome using SILAC. For TbVdu1, ISG65 and ISG75 are the only significantly modulated proteins, but for TbUsp7 a cohort of integral membrane proteins, including the acid phosphatase MBAP1, that is required for endocytosis, and additional ISG-related proteins are down-regulated. Furthermore, we find increased expression of the ESAG6/7 transferrin receptor and ESAG5, likely resulting from decreased endocytic activity. Therefore, multiple ubiquitylation pathways, with a complex interplay with trafficking pathways, control surface proteome expression in trypanosomes.

## Author Summary

The mechanisms by which pathogens interact with their environment are of major importance, both for fulfilling the basic needs of the parasite and understanding immune evasion. For African trypanosomes, the surface is dominated by the variant surface glycoprotein (VSG), but recent data has demonstrated an important role for ubiquitylation in mediating turnover of invariant surface glycoproteins (ISGs) and maintaining ISG copy number independent of VSG. Further, ISG expression is required for suramin-sensitivity. Here we describe mechanisms mediating ISG turnover, uncovered using a screen for genes involved in sensitivity to suramin. These involve multiple aspects of the ubiquitylation machinery, and connect ISG turnover with additional surface proteins. Our data provide a first insight into the complexity of regulation of the ISG family, identifying further aspects to the control of a drug-sensitivity pathway in trypanosomes, and offering insights into metabolism of the parasite surface proteome.

## Introduction

*Trypanosoma brucei* is the causative agent of human African trypanosomiasis (HAT) and nagana, and severely impacts both human health and economic prosperity in sub-Saharan Africa. HAT is subdivided into two forms; acute caused by the *T*. *b*. *gambiense* subspecies and chronic caused by *T*. *b*. *rhodesiense*. Currently, five drugs are available to treat HAT and, while deployment depends on disease stage and subspecies, adverse toxicity, complex administration regimes and emerging resistance all contribute to the need for new therapies and improved understanding of the mechanisms by which existing drugs act [1]. Recent appreciation of *T*. *evansi* and *T*. *equiperdum* as very closely related to *T*. *brucei* extends the impact of the African trypanosomes to much of Asia and Latin America [2].

Bloodstream-form trypanosomes exhibit a highly efficient endocytic system that enables rapid recycling of surface proteins, antibody clearance and nutrient uptake. This is reflected by the presence of the flagellar pocket, a defined membrane region at the flagellar base, dedicated to incoming and outgoing membrane traffic [3]. This organelle facilitates rapid uptake and recycling of variant surface glycoproteins (VSGs), dimeric, glycosylphosphatidylinositol (GPI) anchored glycoproteins that dominate the cell-surface at this life cycle stage. The dense VSG surface coat, that has the ability to undergo antigenic variation by switching between immunologically distinct VSG variants, is recognized as the primary defence against both, innate and acquired immune response [4].

Intercalated with the VSG-coat are *trans-*membrane-domain (TMD) proteins. Invariant surface glycoprotein (ISG) families are amongst the most abundant TMD-proteins of bloodstream form *T*. *brucei* [5,6], with ISG65 and ISG75 estimated at 70,000 and 50,000 copies per cell, respectively [7]. Significantly, both these type I TMD proteins are modified by ubiquitylation, with internalisation and degradation depending on ubiquitylation at specific cytoplasmic residues [7,8]. In higher eukaryotes, ubiquitylated proteins destined for the lysosome are regulated by the endosomal sorting complex required for transport (ESCRT) machinery, where ubiquitylated proteins are recognised and sorted into multivesicular bodies (MVBs) [9]; similar pathways probably operate in trypanosomes [10].

Recent studies linked ISG75 to the action of suramin [11], the oldest trypanosome drug remaining in the pharmacopeia, but which is only useful in the clinic against early stage *T*. *b*. *rhodesiense* [12]. Using genome wide RNAi-target sequencing we identified a cohort of genes involved in sensitising trypanosomes to suramin [11]. This implicated, along with ISG75, multiple proteins with roles and/or locations at the endocytic pathway, including a major facilitator superfamily transporter (MFST) [13], two deubiquitylating enzymes (DUBs) orthologous to human Usp7 and Vdu1, the AP-1 adaptin complex [14], Golgi/lysosomal protein-1 (GLP-1) [15], the trypanosome ortholog of Vps5 [16], cathepsin-L (CatL) [17,18] and the essential lysosomal protein p67 [19,20]. Specific knockdowns of several of the above, including the two DUBs, significantly increased suramin resistance ([11] and S1 Fig). The absence of many central endocytic genes, e.g. clathrin, Rab5 and Rab7, from the suramin sensitivity gene cohort likely arises from the severe lethality that knockdown of these genes elicits.

AP-1 is involved in bi-directional clathrin-dependent transport between the *trans*-Golgi network (TGN) and endosomes in higher eukaryotes [21], and while its role in *T*. *brucei* is less clear, AP-1 does participate in trafficking of lysosomal protein p67 in bloodstream form trypanosomes, potentially connecting these suramin-sensitivity genes [14,22]. Further, the absence of the AP-2 complex in *T*. *brucei* suggests that AP-1 likely assumes a more prominent endocytic role in African trypanosomes [23]. Significantly, no cargo-specific DUBs are known in trypanosomatids, but it is possible that ISG75 and the DUBs identified as suramin-sensitivity determinants are in some manner connected. Suramin most likely gains access to the cytoplasm *via* the MFST family of transporters, to interfere with additional cellular functions, including glycolysis [24,25].

Our current model is that suramin binds to ISG75 and is delivered *via* endocytosis to the lysosome, where CatL releases suramin from its binding partner [26]. However, definitive evidence for a direct interaction with ISG75 and a role for ISG75 as the suramin receptor remains to be obtained. Further, we have not integrated the role of the AP-1 complex into our model. We previously demonstrated that TbUsp7 knockdown specifically decreased ISG75, but not ISG65, expression levels. Given the similarities in architecture, trafficking, stage-specific expression and evolutionary histories, these findings suggest a complex mechanism underpinning ISG expression, and thus suramin sensitivity.

To define the pathways affecting these processes, we examined the roles of TbUsp7, TbVdu1 and TbAP-1 on ISG trafficking, combining knockdown, imaging, analysis of ISG turnover, as well as global proteome analysis. We find a complex interaction between ISGs and deubiquitylation pathways, as well as connections to additional surface proteins, indicating connectivity between ISG trafficking, endocytosis and nutrient acquisition. These data, in conjunction with our earlier findings [11], also demonstrate that sensitivity of bloodstream-form trypanosomes to suramin is dependent on their uniquely active endocytic apparatus, and further confirms that exploitation of this pathway has significant potential for the development of novel therapeutics.

## Results

### AP-1 is not required for either localisation or stability of suramin-sensitivity gene products

**Fig 1:**
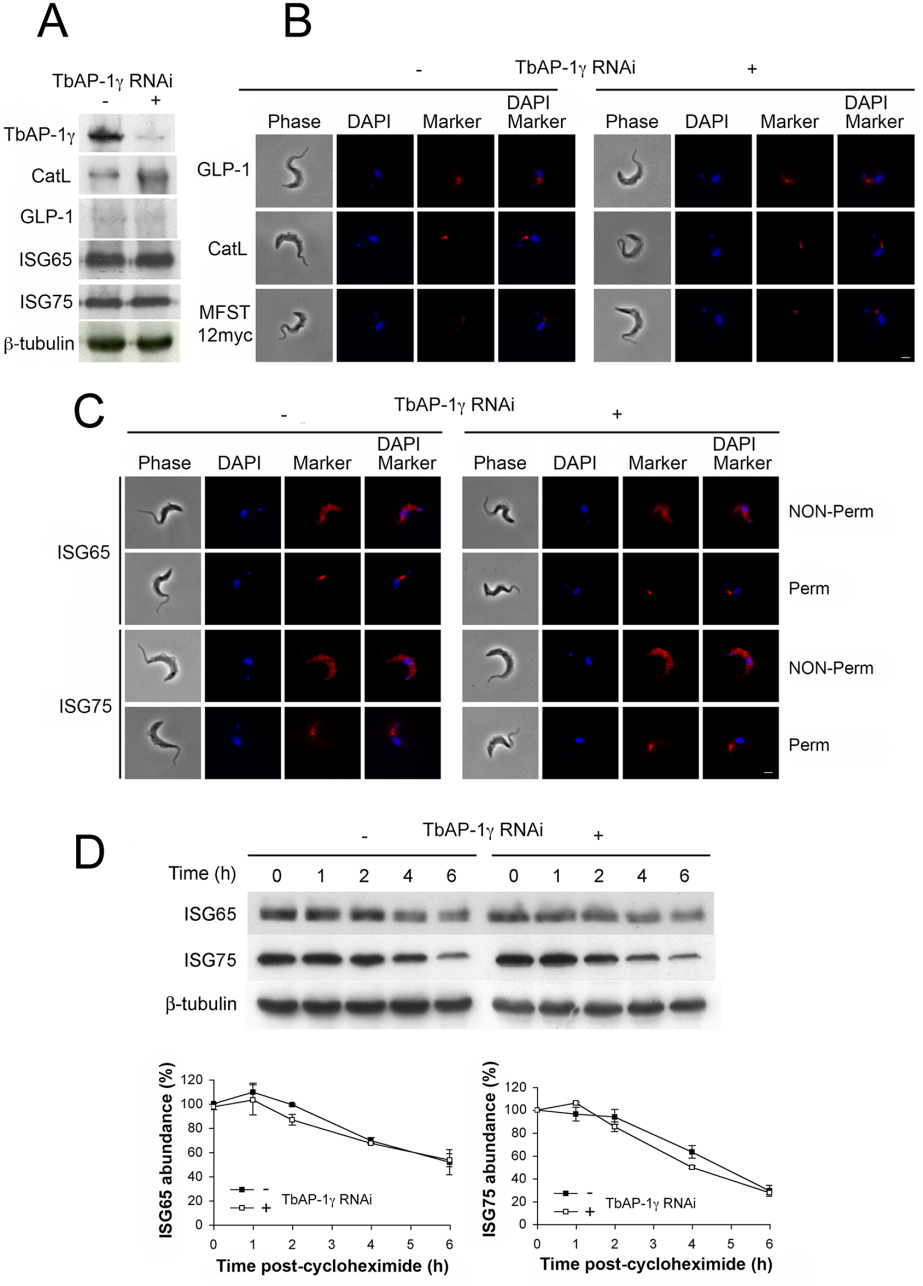
The effect of AP-1γ RNAi on markers of the endocytic pathway. All experiments were performed in uninduced (-) or induced (+) cells for 18 hours. (A) Western blot and (B) immunofluorescence for GLP-1, CatL, MFST^12myc^, (C) ISG65 and ISG75. In B and C scale bar = 2.5 μm, protein antigens are shown in red, DNA visualised with DAPI is in blue. (D) AP-1γ RNAi cells were subjected to RNAi knockdown followed by cycloheximide treatment. Cells were harvested at various time points and endogenous ISG65 and ISG75 levels were monitored by western immunoblotting. β-tubulin was used as a loading control. Lower panel shows quantification, with open symbols induced (+) and closed symbols uninduced (-) and representing the mean of three independent experiments, with the standard error indicated.

All four subunits of the trafficking adaptor, AP-1, were identified in our screen for suramin-sensitivity. Given the known roles for this complex in post-Golgi trafficking in higher eukaryotes, we first asked if trafficking and stability of proteins located at the surface or within the endosomal system identified in the suramin screen are regulated by AP-1 [11]. Since knockdown of any AP-1 subunit destabilises the remaining proteins in the complex [14], steady state levels of GLP-1, CatL and MFST were assessed following AP-1γ knockdown [14]. RNAi strongly decreased AP-1γ protein levels (~90%), but no significant changes in steady state levels of GLP-1 were found and only a small increase in CatL level was seen (Fig 1A). As we were unable to obtain reliable data for MFST^12myc^ by Western blotting, we used immunofluorescence (IF) to monitor MFST localisation and copy number. The locations of GLP-1, CatL and MFST were unaffected by AP-1 knockdown, with no significant differences in signal intensity between uninduced and induced cells, suggesting that neither trafficking nor expression levels of these proteins depends significantly on AP-1. AP-1 knockdown also had no obvious effect on ISG65 and ISG75 localisation (Fig 1C), steady state levels or turnover (Fig 1A and 1D).

We conclude that ISG65 and ISG75 are either targeted by an AP-1-independent mechanism or that a redundant pathway assumes the role of AP-1 in its absence. It is likely that the absence of a major effect on ISG reflects the specific roles of AP-1. Significantly, another suramin sensitivity determinant, the lysosomal membrane protein p67, depends on AP-1 for its lysosomal delivery. Evidence suggests that trafficking of p67 is *via* a distinct route from ISGs that does not involve transport through the flagellar pocket. Moreover, p67 trafficking is reliant on classical dileucine motifs within the cytoplasmic domain. Hence, the identification of the AP-1 complex as a suramin sensitivity determinant probably reflects a role in the trafficking of p67 or other lysosomal proteins required to maintain the lysosomal environment necessary for suramin action [11, 14, 20, 22, 27].

### Suramin-sensitivity DUBs are evolutionarily conserved

The suramin-sensitivity screen identified two deubiquitylating enzymes, encoded by Tb927.9.14470 and Tb927.11.12240. *In silico* analysis indicated that both are evolutionarily conserved across eukaryotes, with the human orthologs being USP7 and VDU1, respectively, and therefore we designated these gene products as TbUsp7 and TbVdu1. Significantly, USP7 is involved in multiple pathways including cell cycle control, epigenetics and the immune response, and is the target of anti-oncogenesis drug screening efforts [28]. Conversely, VDU1 is predicted as membrane-associated and part of a complex ubiquitylation switch controlling expression of the HIF-1α transcription factor [29]. VDU1 exists in a complex with an Rbx/cullin/elongin E3 ubiquitin ligase that provides a rapid mechanism for downregulation of receptor tyrosine kinases upon ligand binding, and orthologs of all components of this E3 ubiquitin ligase are present in the *T*. *brucei* genome, suggesting that trypanosomes possess an analogous complex.

### TbUsp7 and TbVdu1 have roles in ISG trafficking

**Fig 2:**
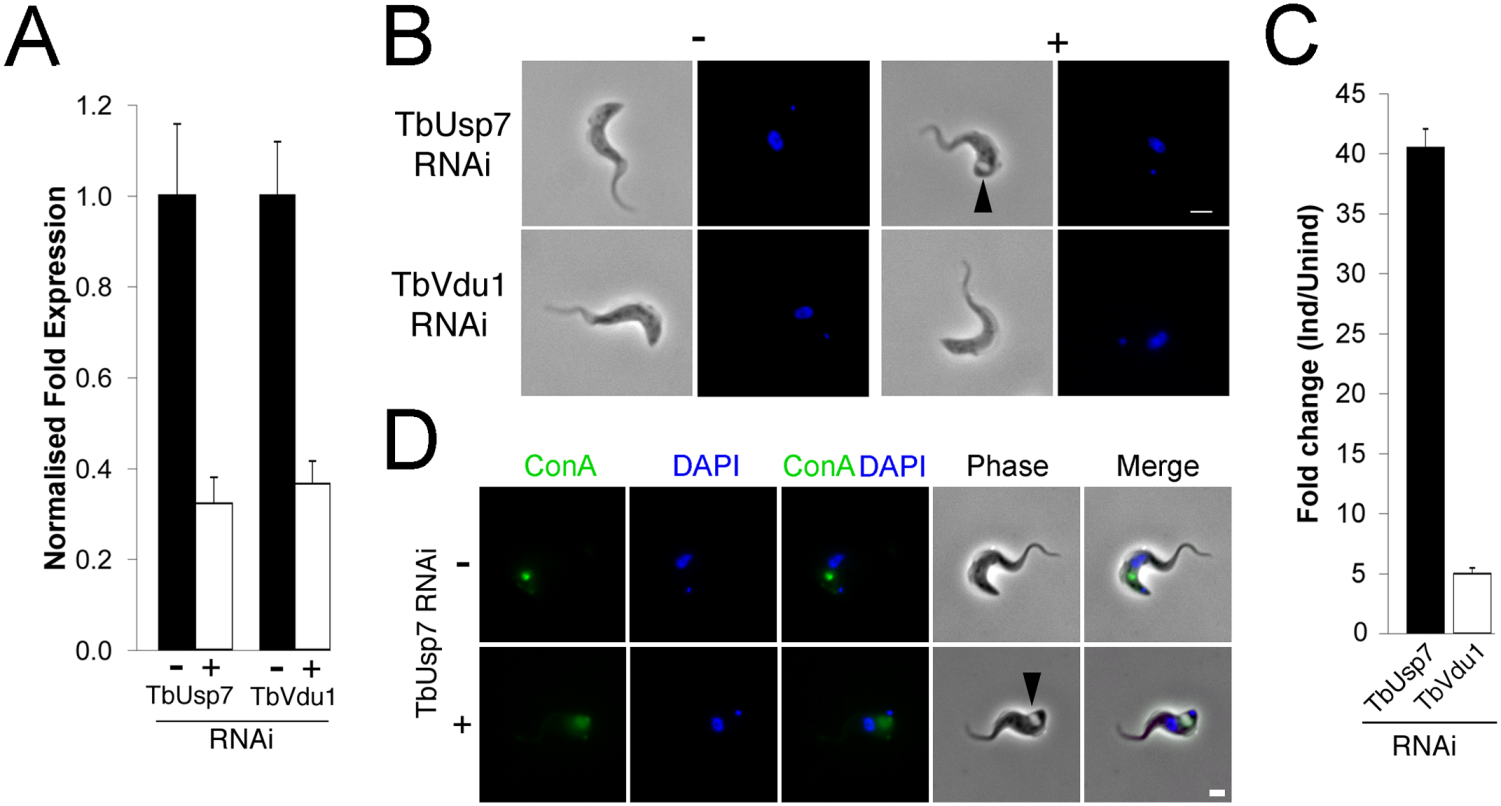
TbUsp7 but not TbVdu1 knockdown perturbs endocytosis. (A) Knockdown of TbUsp7 and TbVdu1 in the presence (+, open bar) or absence (-, closed bar) of RNAi induction as measured by qRT-PCR. Results were normalised to β-tubulin and error bars denote the standard error of the mean (n = 3). (B) Phase contrast images showing the presence or absence of the BigEye phenotype under TbUsp7 and TbVdu1 knockdown. The enlarged flagellar pocket is indicated by a black arrowhead. Scale bar = 2.5 μm. (C) One hundred cells were analysed from uninduced or induced cultures of TbVdu1 (open bar) or TbUsp7 (closed bar) and scored for the appearance of an enlarged flagellar pocket, manifest as a phase-light vacuole at the posterior end of the cell. Results represent an average of four independent experiments with error bars denoting the standard error. (D) ConA uptake in the presence and absence of TbUsp7 knockdown. Following induction of RNAi, cells were incubated with FITC-conjugated ConA (green). Note that only an example of a cell with an enlarged flagellar pocket is presented for TbUsp7. Induced cells that do not display this morphology have normal ConA uptake. Scale bar = 2.0 μm. All cells were co-stained with DAPI (blue) to visualise nuclear and kinetoplast DNA.

Cells harbouring stem-loop RNAi constructs specific for either TbUsp7 or TbVdu1 were induced with tetracycline and their mRNA abundance assessed by qRT-PCR; both were silenced by over 60% (Fig 2A). We previously observed growth defects for TbUsp7 under these conditions [11], but in contrast, knockdown of TbVdu1, a probable membrane-associated protein confined to the flagellar pocket region, did not impact proliferation (S1 Fig). Furthermore, while knockdown of TbVdu1 did not induce morphological defects at the light microscopy level, knockdown of TbUsp7 led to enlargement of the flagellar pocket, the ‘BigEye’ phenotype originally observed for knockdown of clathrin, and consistent with the proliferative defect resulting from TbUsp7 RNAi (Fig 2B and [30]). Quantification revealed that ~20% of cells in TbUsp7 knockdown cultures possess the BigEye morphology, constituting a 40-fold increase in BigEye frequency, compared with ~5-fold increase for TbVdu1 knockdown (Fig 2C).

As enlargement of the flagellar pocket suggests an endocytic block, we monitored endocytosis with fluorescent-ConA in TbUsp7 and TbVdu1 knockdown cells. ConA rapidly enters into early endosomes and is subsequently delivered to the lysosome in trypanosomes, a process unaffected by TbVdu1 knockdown (S2 Fig). In contrast, TbUsp7 knockdown blocked ConA uptake in BigEye cells (Figs 2D and S2), with ConA remaining at the flagellar pocket over the course of the assay. TbUsp7 knockdown cells without the BigEye phenotype displayed ConA trafficking similar to uninduced cells, confirming that the BigEye morphology was indeed associated with blocked endocytosis. While the kinetics of appearance of the BigEye morphology was significantly slower than following clathrin knockdown, it was similar to knockdown of several other proteins acting within the endosomal apparatus, for example Rab5 [31] and TbCAP116 [32], suggesting that modification of a critical component of the endosomal apparatus is impacted by TbUsp7 knockdown or that the protein has some direct role in endocytosis. We therefore asked whether TbUsp7 knockdown impacts clathrin itself, as Rab5 knockdown leads to a BigEye phenotype *via* decreased expression of clathrin [31]. However, steady state levels of clathrin were unaffected (S3 Fig), suggesting another molecular target.

### Knockdown of clathrin leads to increased ISG75 ubiquitylation

**Fig 3:**
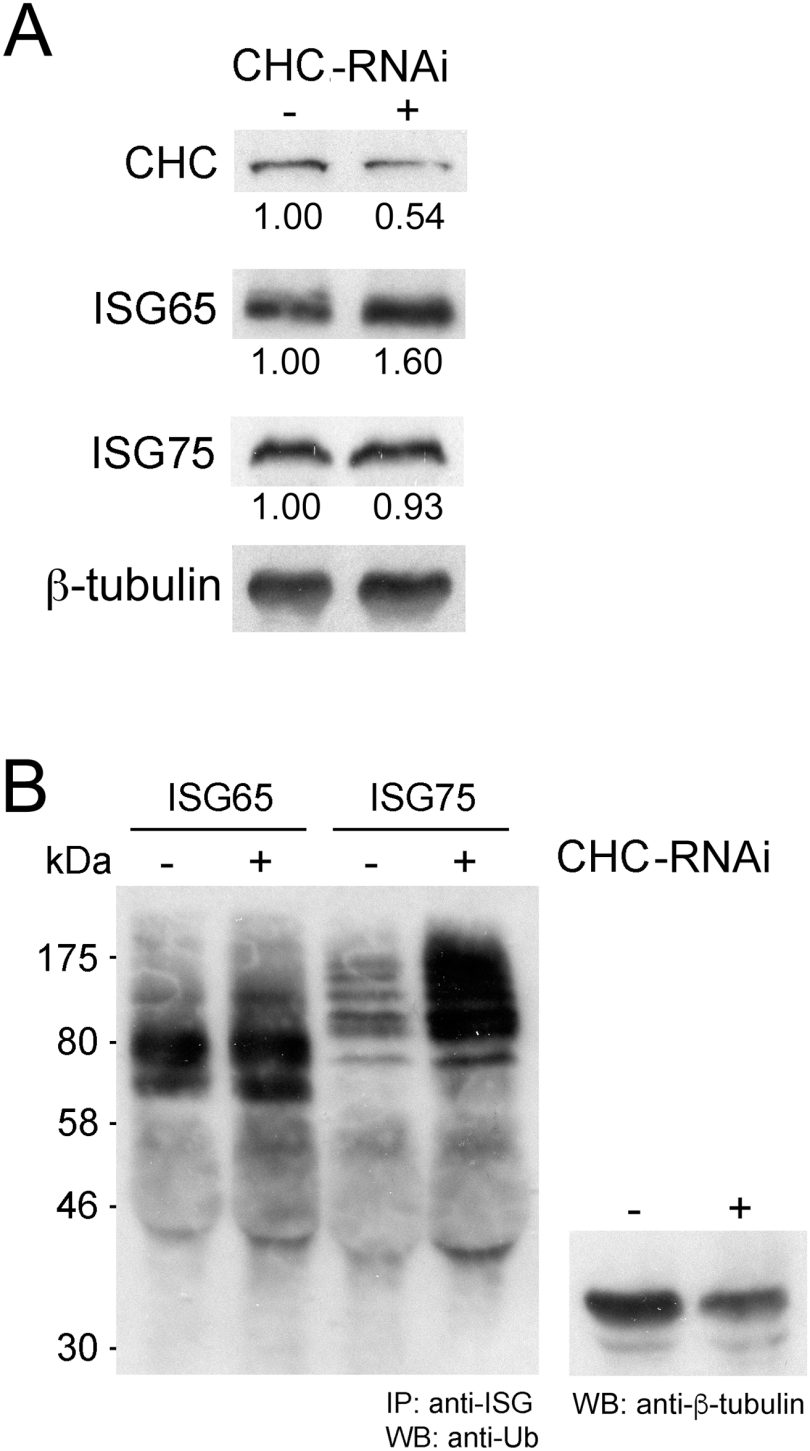
Clathrin knockdown increases ubiquitylation of ISG75. (A) Levels of clathrin heavy chain (CHC), ISG65 and ISG75 were monitored following RNAi knockdown of clathrin for 12 hours by western immunoblotting. β-tubulin was used as loading control and relative protein abundance was quantified by densitometry. (B) Endogenous ISG65 and ISG75 were immunoprecipitated using specific antibodies followed by western immunoblotting with anti-ubiquitin antibody. A western immunoblot from the same samples prior to immunoprecipation and probed with anti-β-tubulin was used as a loading control.

ISG65 and ISG75 are both ubiquitylated *in vivo*, but the location where this modification occurs is unknown. To determine if ubiquitylation required entry to the cell interior or could occur at the cell surface, we performed RNAi knockdown of clathrin and monitored both steady state expression and the ubiquitylation status of ISG65 and ISG75. Clathrin knockdown results in decreased intracellular ISG65 with a concomitant increase in steady state levels of ISG65 (Fig 3A), consistent with a block in endocytosis and degradation [7]. By contrast, clathrin knockdown had no effect on steady state levels of ISG75 (Fig 3A). Furthermore, while we did not observe a significant difference in the ubiquitylation profile of ISG65 in clathrin knockdown relative to uninduced cells, ISG75 consistently showed increased ubiquitylation (Fig 3B). As endocytosis has been blocked, this indicates that ISG75 is likely ubiquitylated at the cell surface and prior to internalisation, but there is no similar evidence for ISG65. Together, these data suggest that while ISG65 and ISG75 are both ubiquitylated, the modification likely occurs at different sub-cellular locations and by distinct mechanisms.

### ISG65 and ISG75 expression levels depend on TbUsp7 and TbVdu1

**Fig 4:**
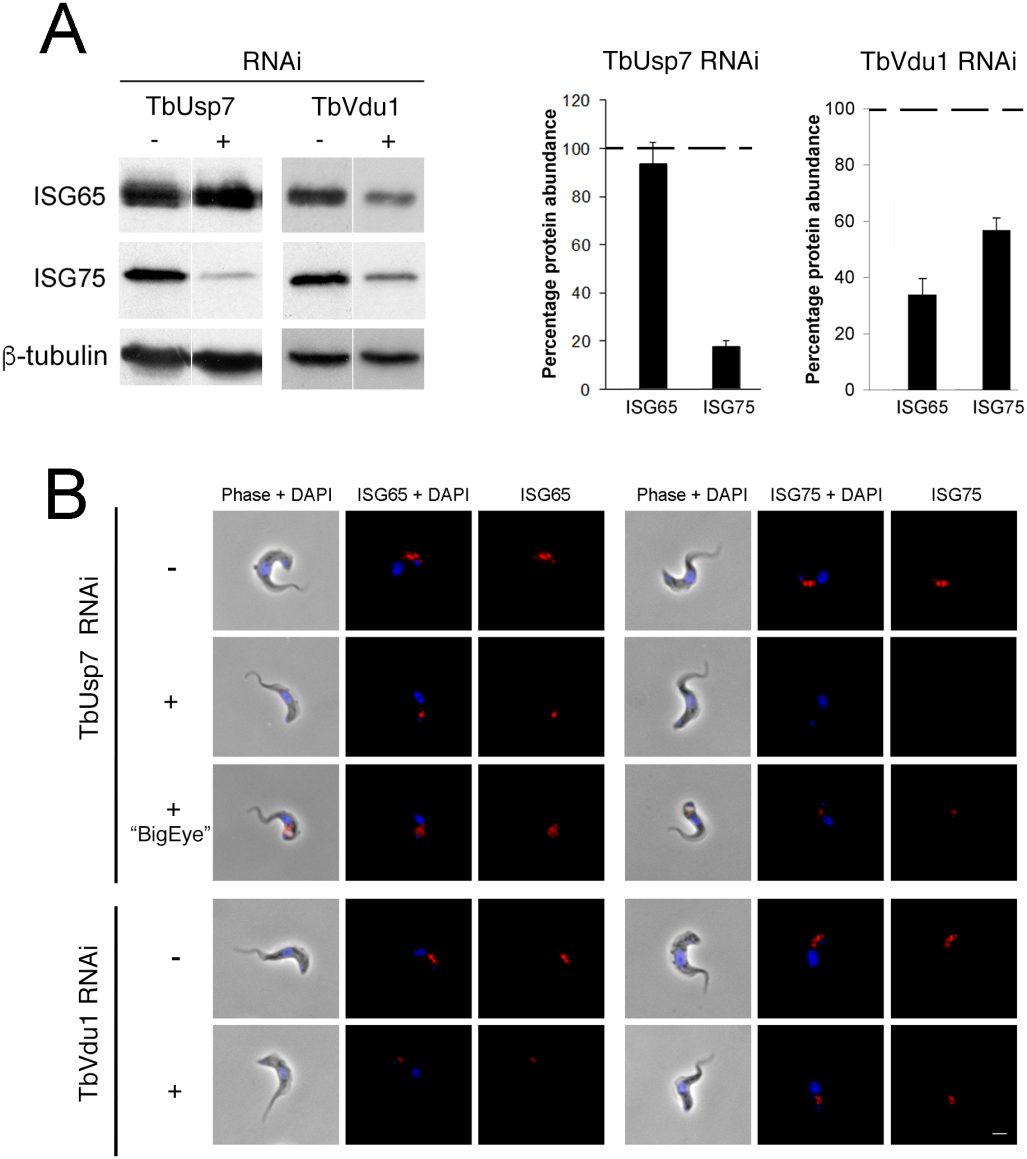
Knockdown of TbUsp7 and TbVdu1 differentially reduces steady state ISG65 and ISG75 levels. (A) Steady state levels of endogenous ISG65 and ISG75 in the presence (+) or absence (-) of TbUsp7 and TbVdu1 knockdown were assessed by western immunoblotting. β-tubulin was used as a loading control. Bar graphs represent the mean of three independent experiments normalised to uninduced cells at 100% (dotted line), with the standard error indicated. (B) The location and levels of ISG65 and ISG75 were assessed by immunofluorescence in the presence or absence of TbUsp7 or TbVdu1 knockdowns (+ or -, respectively). Cells with enlarged flagellar pockets (“BigEye”) in the presence of TbUsp7 RNAi are also shown. Cells were stained with DAPI (blue) to visualise nuclear and kinetoplast DNA, while ISG65 and ISG75, visualised with specific polyclonal antibodies, are shown in red. Scale bar = 2.5 μm.

We next assessed expression levels of ISG65 and ISG75 following knockdown of the two DUBs. TbUsp7 knockdown resulted in strongly decreased ISG75 expression (to ~20%) but not ISG65, as previously shown [11]. Significantly, TbVdu1 knockdown led to decreases in both ISG75 (to ~60%) and ISG65 (to ~35%) (Fig 4A).

These observations were corroborated by immunofluorescence microscopy, where intracellular pools of ISG75, but not ISG65, were significantly diminished by TbUsp7 knockdown, while both ISG65 and ISG75 were reduced on TbVdu1 knockdown (Fig 4B). Where a residual intracellular signal was observed in TbVdu1 knockdown cells, ISG65 and ISG75 remained localised to posterior endomembrane compartments, and hence were not significantly mislocalised. However, in cells exhibiting the BigEye phenotype following TbUsp7 knockdown, ISG65 and ISG75 staining accumulated at the enlarged flagellar pocket, presumably retained due to blocked endocytosis (Fig 4B). Overall, these data suggest that while ISG65 and ISG75 are not mistargeted, decreased expression level represents the primary impact of TbUsp7 or TbVdu1 knockdown.

### TbUsp7 and TbVdu1 control ISG turnover

**Fig 5:**
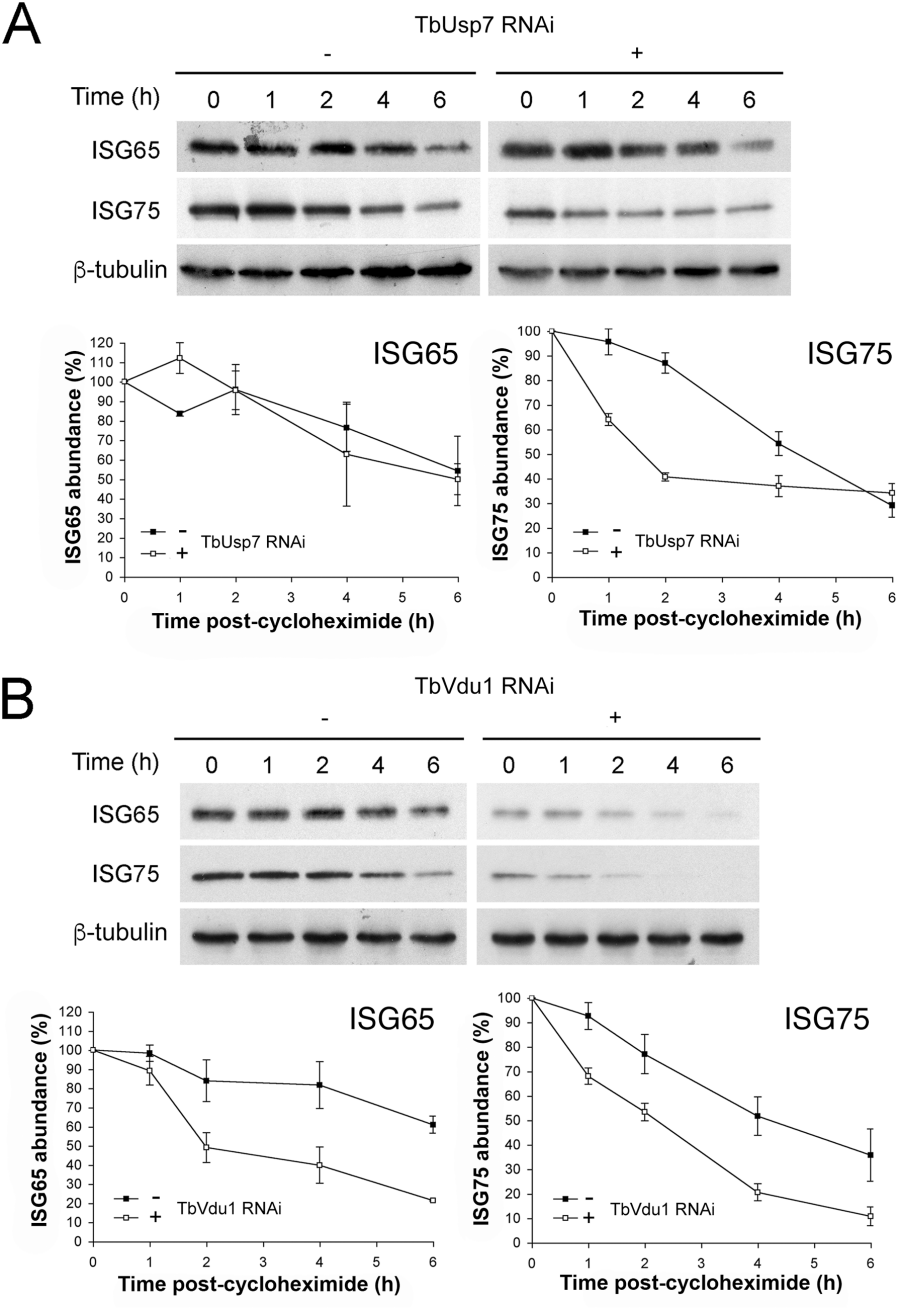
ISG65 and ISG75 turnover under TbUsp7 and TbVdu1 knockdown. (A) TbUsp7 and (B) TbVdu1 RNAi knockdown was followed by cycloheximide treatment. Cells were harvested at various time points and endogenous ISG65 and ISG75 levels were monitored by western immunoblotting. β-tubulin was used as a loading control. Closed symbols indicate uninduced (-) and open symbols induced (+) cultures. Graphs represent the mean of three independent experiments, with the standard error indicated.

We next examined degradation rates for ISG65 and ISG75 in DUB knockdowns by blocking protein synthesis with cycloheximide and measuring protein abundance by Western blotting. While ISG75 was significantly destabilised, ISG65 degradation was unaffected by TbUsp7 knockdown (Fig 5A). In contrast, both ISG65 and ISG75 were turned over more rapidly in TbVdu1 knockdown cells (Fig 5B). All of these observations indicate that the major mechanism underpinning lower ISG65 and ISG75 expression levels is decreased stability/increased turnover in DUB knockdown cells, consistent with an inhibitory effect of DUBs on ISG ubiquitylation levels.

### Effect of DUB knockdown on ISG transcript and de novo synthesis

**Fig 6:**
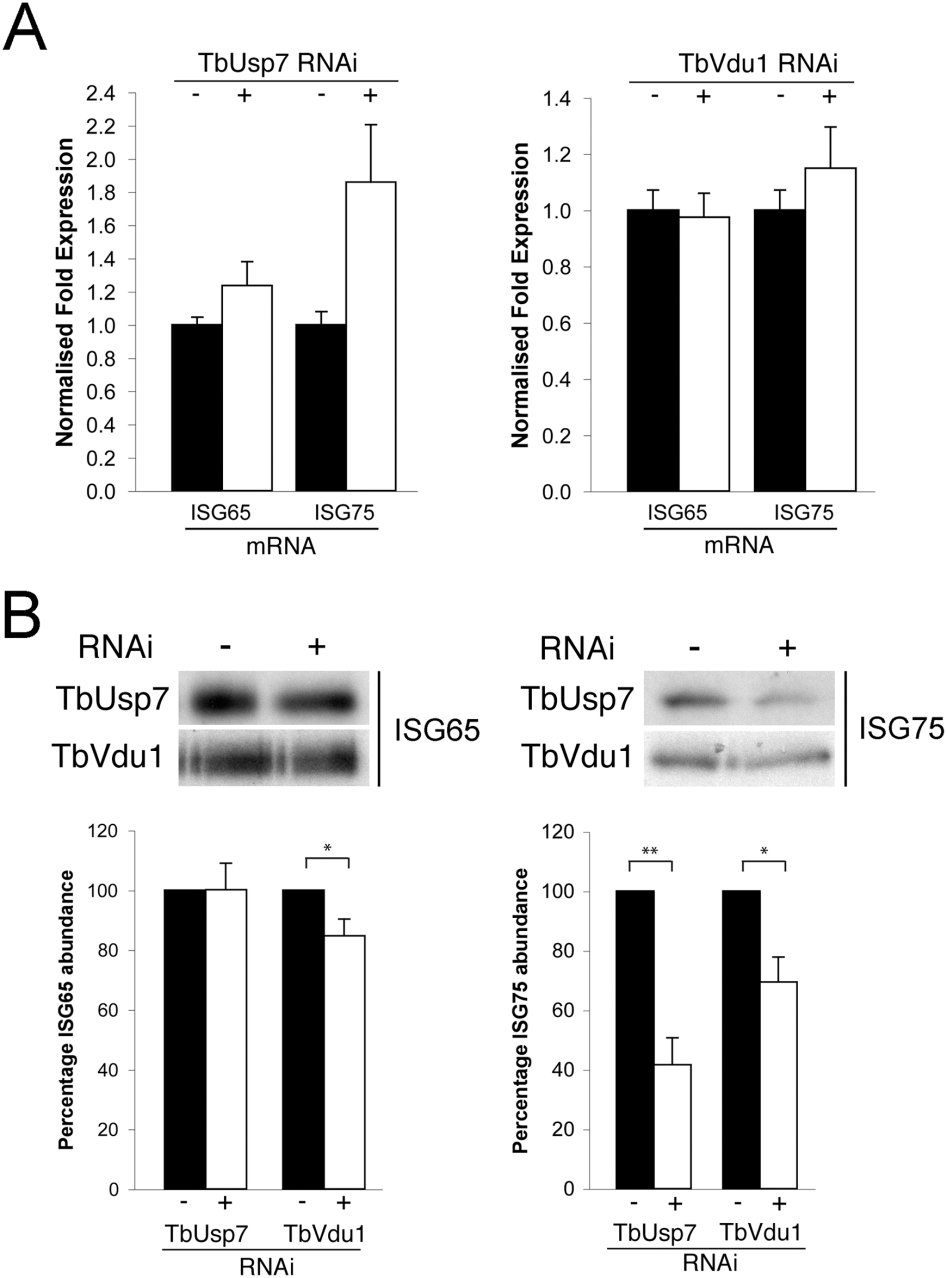
Assessment of ISG65 and ISG75 transcription and biosynthesis under TbUsp7 and TbVdu1 knockdown. (A) ISG65 and ISG75 transcript levels were determined in the presence (+, open bars) or absence (-, closed bars) of TbUsp7 and TbVdu1 knockdown by qRT-PCR normalised to β-tubulin. Error bars denote standard error of the mean. (B) Biosynthesis of ISG65 and ISG75 were monitored by immunoprecipitation using specific polyclonal antibodies, in the presence (+) or absence (-) of TbUsp7 and TbVdu1 knockdown. Bar graphs represent the mean of three independent knockdown experiments (open bars) normalised to uninduced (closed bars) cells, with the standard error indicated. Statistical analysis: Student’s t-test; *p<0.05, **p<0.01.

To determine if the impact of DUB knockdown on ISG expression was due to changes in synthesis or turnover, we initially quantified the respective mRNAs by qRT-PCR. While no significant differences were observed in transcript levels for either ISG when TbVdu1 was knocked down, we observed almost twice as much ISG75 mRNA in the presence of TbUsp7 knockdown compared with uninduced cells (Fig 6A). This suggests the presence of a feedback mechanism able to upregulate ISG75 transcript levels in order to maintain copy number upon protein destabilisation. The lack of evidence for decreased ISG mRNA levels in either DUB knockdown, indicates that reduced transcription can be excluded as an explanation for the observed decrease in ISG protein level.

To examine if decreased translation could account for lower ISG expression level following DUB knockdown, cells were pulse-labelled for one hour with ^35^S-methionine followed by immunoprecipitation of ISG65 and ISG75. We observed a small effect on ^35^S-methionine incorporation into ISG65 following TbVdu1 depletion, but this was substantially less than the observed destabilisation. We saw more pronounced decreased ^35^S-methionine incorporation into ISG75 following TbUsp7 (~60% decrease) or TbVdu1 (~30% decrease) knockdown (Fig 6B). While we cannot exclude that ISG75 biosynthesis is affected by DUB knockdown, given that the cells were pulse-labelled for 60 minutes and ISG75 is turned over rapidly following DUB knockdown (T_1/2_ 1.5 and 2.0 hours following TbUsp7 and TbVdu1 knockdown, respectively, Fig 5A and 5B), accelerated turnover likely accounts for the majority of the decrease in ISG75 labelling, as a substantial portion of the labelled ISG75 would have been degraded even during the ^35^S-pulse period. Taken together, these data indicate that the majority of observed changes in ISG65 and ISG75 expression following DUB depletion likely arise from accelerated turnover rather than biosynthesis, suggesting that the DUBs influence ISG modification directly.

### The cytoplasmic domain of ISG75 is sufficient for TbUsp7-mediated destabilisation

As knockdown of TbUsp7 and TbVdu1 has such a profound effect on ISG75 expression, we were unable to monitor the ubiquitylation status of ISG75 under these conditions, as even in wild type cells ubiquitylated adducts of ISG75 are a very minor fraction, and attempts to detect such adducts in TbUsp7 knockdowns were unsuccessful. As an alternative approach, we reasoned that if TbUsp7 was acting directly on ISG75, then a reporter construct that bore the ubiquityation sites, but lacked the ISG75 ectopic domain would behave in a similar manner to ISG75 in TbUsp7 knockdown cells.

**Fig 7:**
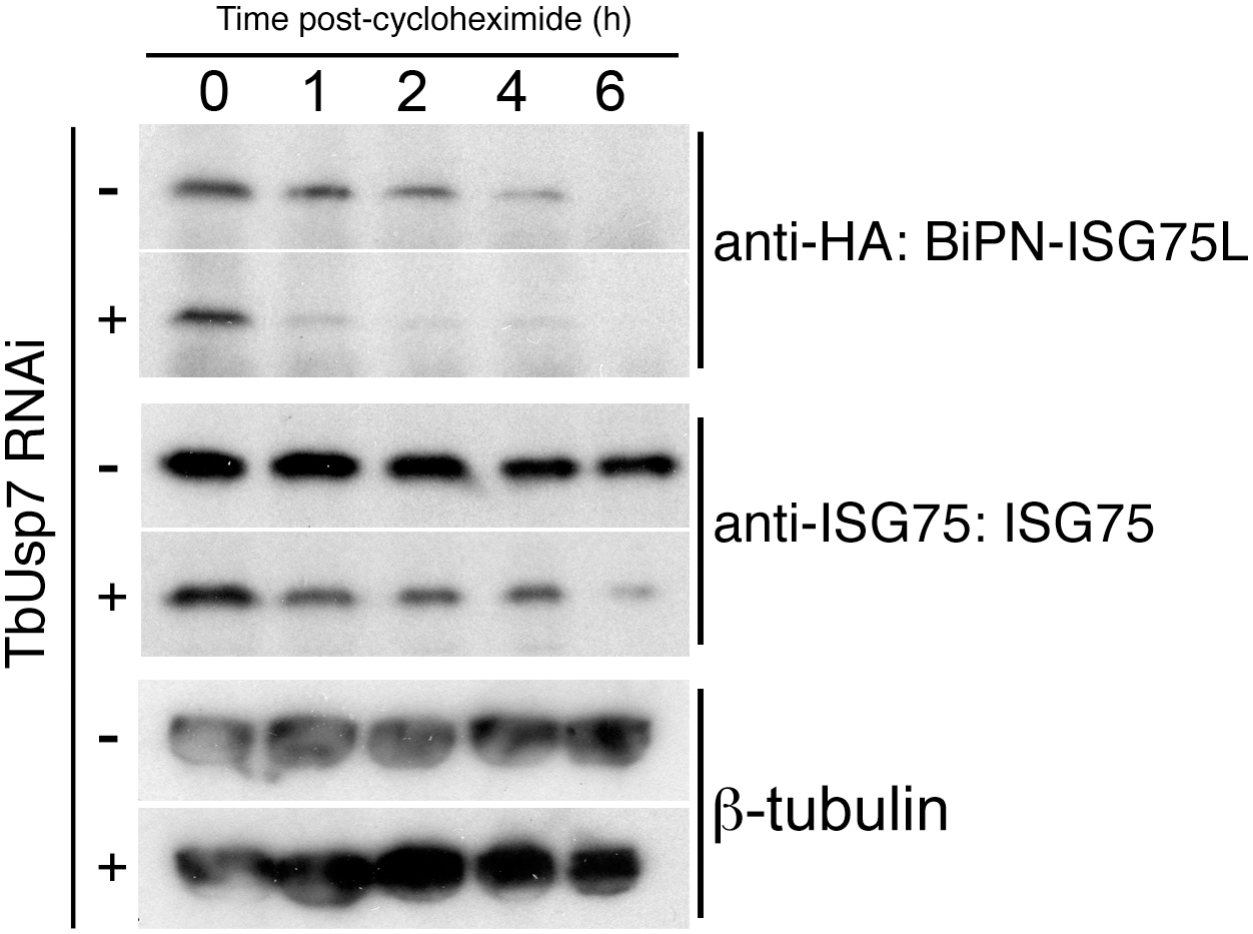
DUB-knockdown mediated expression level changes depend on cytoplasmic domain sequences. BiPN-ISG75L is a chimeric reporter construct containing the BiP ATPase N-terminal domain fused to an HA epitope tag followed by the *trans-*membrane and cytoplasmic domain of ISG75. Cells were grown in the presence (+) or absence (-) of TbUsp7 RNAi for 48 hours followed by treatment with cycloheximide for the indicated times. The levels of HA-BiPN-ISG75L and endogenous ISG75 were assessed at different time points by western immunoblotting with anti-HA or anti-ISG75 antibodies. β-tubulin was used as a loading control.

TbUsp7 RNAi cells were transfected to overexpress BiPN-ISG75L, a reporter containing the BiP ATPase N-terminal domain (BiPN) fused to an HA-tag and followed by the *trans-*membrane and cytoplasmic domain of ISG75 [8]. As before, we observed an increased destabilisation of ISG75 both at steady state (at time 0 h) and over time following TbUsp7 knockdown, and also increased turnover of BiPN-ISG75L relative to uninduced cells (Fig 7). These data indicate that the cytoplasmic domain of ISG75 is sufficient for TbUsp7-mediated stabilisation, and suggests that modulation of ubiquitylation likely accounts for altered ISG75 expression levels.

### TbVdu1 and TbUsp7 differentially interact with the trypanosome proteome

To extend our understanding of interactions between TbUbp7 and TbVdu1 and the parasite surface, we analysed the effect of DUB knockdown on the whole cell proteome using stable isotope-labeling by amino acids in culture (SILAC, see methods). As maximal knockdown for both DUBs was achieved by 48 hours, we examined cells at 26 and 48 hours after induction for TbUsp7, and at 48 hours for TbVdu1 knockdown. We were able to quantify 1769, 1823 and 1879 distinct proteins from these analyses respectively, representing approximately 25% of the total cellular proteome. As the vast majority of proteins were unaltered, this indicates that the detected changes are specific and not the result of general toxicity arising from the knockdown.

**Fig 8:**
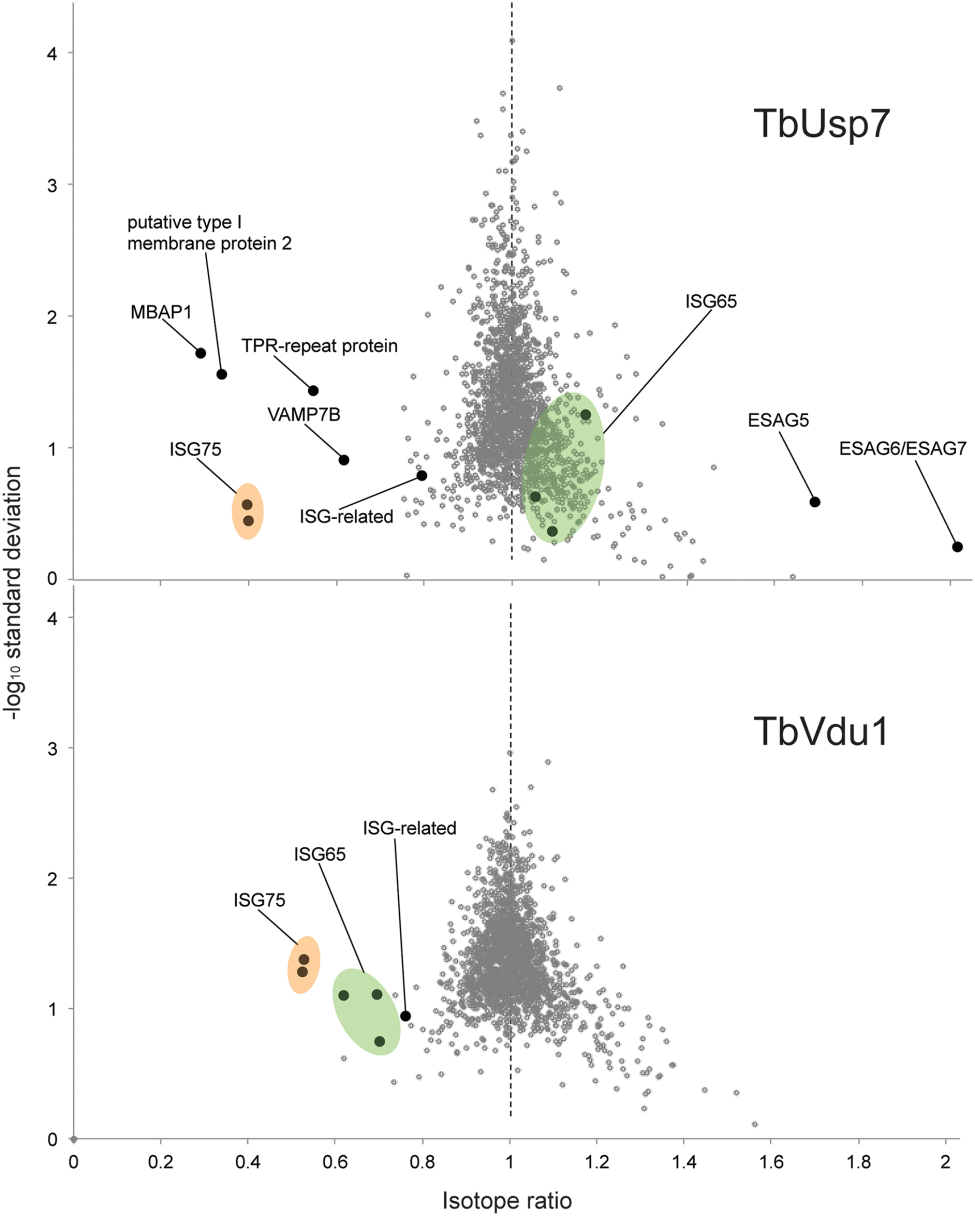
Volcano plots of protein abundance changes. Normalised SILAC ratios, averaged from duplicates (TbUsp7) or triplicates (TbVdu1), are plotted against the respective -log10 transformed standard deviation. Data points representing protein groups significantly shifted after 48 hours are labeled (see also Table 1). ISG65 and ISG75 paralogs are highlighted in green and orange, respectively.

The impact of TbUsp7 and TbVdu1 on ISG65 and ISG75 determined by SILAC (see Table 1, Fig 8) was in perfect agreement with semi-quantitative Western blotting (see above). TbUsp7 knockdown reduced ISG75 abundance to ~40% after 48 hours, whilst total ISG65 levels were unchanged. TbVdu1 knockdown decreased ISG75 and ISG65 to ~55% and ~65% after 48 hours, respectively. ISG75 paralogs were equally affected by the DUB knockdowns, and similarly the ISG65 paralogs were also decreased uniformly. Furthermore, SILAC analyses revealed an impact of TbVdu1 knockdown on additional proteins, including a distant ISG paralog, Tb927.5.630, that was also decreased.

**Table 1:** Percentage abundance of selected protein groups upon TbUsp7 and TbVdu1 RNAi derived from normalised SILAC ratios. Values represent average percentage protein abundance relative to uninduced cells ± standard deviation. TbUsp7 and TbVdu1 RNAi samples were analysed at indicated time points in experimental duplicate and triplicate, respectively. Asterisks mark proteins not quantified in all replicates. Polypeptide features predicted using TMHMM2 for *trans-*membrane domains [39], signalP for N-terminal ER-targeting signal [40], PredGPI for GPI-anchor addition C-terminal signal sequence [41] and TPRpred for tetratrico peptide repeats [42]. Predictions are given as Yes, No responses or sequence positions (inclusive signal sequence) using default parameters. In most cases the topology of the protein is experimentally known or predictable based on close homology of experimentally derived information. All protein hits shown have been inspected for their genomic context and integrity. Protein groups consist of indistinguishable paralogs, sharing identical quantified peptides. For the complete quantification data see S1 Table. NA; not applicable, ND; not detected.

Annotation	Protein group	Protein abundance upon RNAi (percent relative to non-induced)			Predicted features	Predicted *Trans-*membrane domain	Predicted N-terminal signal	Number of lysines in cytoplasmic domain	Predicted GPI-anchor	Sequence length (inclusive signal seq.)
		TbUsp7 26h	TbUsp7 48h	TbVdu1 48h						
USP7	Tb927.9.14470	28 *	13 *	101 (+/- 3)	USP7_C2 superfamily	No	No	NA	NA	1161
VDU1	Tb927.11.12240	ND	105 *	ND	*Peptidase_C19 superfamily*	No	No	NA	NA	790
ISG75	Tb927.5.390	43 (+/- 4)	40 (+/-34)	52 (+/- 5)	ISG65-75 superfamily	468–490	Yes	5	NA	522
	Tb927.5.400					468–490		4		522
	Tb927.5.350					468–490		4		522
	Tb927.5.360	42 (+/- 3)	39 (+/-26)	53 (+/- 4)	ISG65-75 superfamily	469–491	Yes	5	NA	523
	Tb927.5.370	ND	ND	64 (+/-23) *	ISG65-75 superfamily	469–491	Yes	5	NA	523
ISG65	Tb927.2.3280	96 (+/- 2)	117(+/-5)	69 (+/- 8)	ISG65-75 superfamily	386–408	Yes	4	NA	436
	Tb927.2.3290					388–410		4		436
	Tb927.2.3300					388–410		4		436
	Tb927.2.3310					388–410		3		436
	Tb927.2.3320	97 (+/- 2)	109(+/-41)	45 (+/- 2)	ISG65-75 superfamily	387–409	Yes	3	NA	437
	Tb927.2.3270	90 (+/- 2)	105(+/-22)	62 (+/- 8)	ISG65-75 superfamily	388–410	Yes	4	NA	436
	Tb11.v5.0231					469–491		5		523
	Tb11.v5.0731					388–410		2		430
ISG-related	Tb927.5.630	75 (+/- 3)	79 (+/-16)	76 (+/- 11)	ISG65-75 superfamily	349–372	Yes	4	NA	401
ISG64	Tb927.5.1390*	92 (+/- 3)	98 (+/- 2)	87 (+/- 19)	ISG65-75 superfamily	376–398	Yes	4	NA	434
	Tb927.5.1410					377–399		4		435
	Tb927.5.1430	97 (+/- 9)	107(+/- 5)	94 (+/- 20)	ISG65-75 superfamily	376–398	Yes	4	NA	434
MBAP1	Tb927.11.13130	50(+/- 1)	29 (+/-2)	101 (+/- 11)	acidic phosphatase [33]	459–481	Yes	2	No	524
putative type I membrane protein 1	Tb927.7.470	38 (+/-1)	32 *	85 (+/- 4)		182–204	Yes	4	No	297
putative type I membrane protein 2	Tb927.9.11480	45 (+/-4)	34 (+/-3)	87 (+/- 2)		512–537	Yes	1	No	561
putative type IV membrane protein	Tb927.11.7550	51 (+/-2)	38	97 (+/- 7)*		49–71,	No	3	No	221
						112–134,				
						141–163,				
						190–212				
VAMP7B	Tb927.5.3560	70 (+/-3)	62(+/-12)	81 (+/-21)	Vesicle-associated membrane protein	184–206	No	No	No	796
TPR-repeat protein	Tb927.11.810	46 *	55 (+/-4)	97 (+/- 4)	Tetratrico peptide repeat [42]	No	No	No	No	216
ESAG5	Tb11.v5.0826	142(+/- 2)	170(+/-26)	132(+/-12)	potential lipid or lipo-polysaccha ride binding [36,37]	No	Yes	NA	No	464
	Tb927.7.6860									480
ESAG6	Tb927.7.3250	132(+/-17)	202(+/-56)	101(+/-43)*	Transferrin receptor	No	Yes	NA	Yes	397
ESAG7	Tb927.7.3260					No	Yes	NA	No	339
ESAG2	Tb927.11.14620	114 (+/- 8)	115 (+/-21)	85(+/- 3)		No	Yes	NA	Yes	458
VSG-related protein	Tb927.7.180	126(+/-17)	139 *	117(+/- 4)*	VSG-related	No	Yes	NA	No	437

Perturbation of the proteome by TbUsp7 knockdown was more extensive, necessitating a kinetic analysis. We considered as significant only those proteins where changes were seen at both sampled time points and also where the magnitude of that change increased between the 26 and 48 hour datasets. Significantly, the membrane-bound histidine acidic phosphatase MBAP1 decreased to 29% at 48 hours. This well-characterised *trans*-membrane protein possesses a topology similar to ISGs and is essential for both endocytosis and exocytosis [33]. Two predicted bitopic membrane proteins and a multi-spanning *trans*-membrane protein, all with unknown function, were decreased to a similar degree. Furthermore, the vesicle associated SNARE protein VAMP7B, which localises to endosomes (Divya Venkatesh and MCF, in preparation) and a TPR-repeat containing protein were also decreased. Finally, a small cohort of bloodstream stage-specific GPI-anchor containing proteins was significantly upregulated following TbUsp7 knockdown. The majority of these are VSG expression site-associated genes (ESAGs). ESAG6 and ESAG7, the transferrin receptor [34,35] increased two-fold (Table 1, Fig 8). ESAG5 and to a much lesser extent ESAG2 also increased; these have recently been shown to be surface or endomembrane system proteins [6]. ESAG5 contains BPI (bactericidal/permeability-increasing protein)/LBP (lipopolysaccharide-binding protein)/PLUNC (palate, lung and nasal epithelium clone)-like domains [36], and based on this architecture is proposed to bind lipid or lipopolysaccharide and have a signalling function [37].

Remarkably specific alterations to the trypanosome proteome result from DUB knockdown. These data explain the endocytosis defect resulting from TbUsp7 RNAi, as downregulation of MBAP1 is known to lead to an enlarged flagellar pocket. Given the topology of MBAP1 and the presence of several lysine residues within the short cytoplasmic region, we speculate that MBAP1 is likely ubiquitylated, and hence TbUsp7 may participate in controlling its expression. Increases to expression of ESAG6/7 probably arise from the endocytosis defect, as trypanosomes precisely regulate expression of the transferrin receptor to maintain iron homeostasis [38].

## Discussion

Maintaining cell surface composition is a vital component of cellular homeostasis. For African trypanosomes this process is also essential to ensure integrity of the VSG monolayer, an aspect of surface biology critical for immune evasion and additional functions encompassing nutrient uptake and sensing. The importance of surface protein expression and trafficking in African trypanosomes is also underscored by the recent finding that ISG75 expression level and the endocytic apparatus are involved in suramin uptake and sensitivity. Understanding this process is fundamental both to revealing the mode of action of suramin and for the consideration of exploitation of this pathway for delivery of drugs into trypanosomes. Furthermore, the process is shown here as a component of mechanisms controlling turnover of surface proteins in trypanosomes.

Several components of the mechanisms involved in setting expression levels of VSG, ISG65, ISG75 and the ESAG6/7 transferrin receptor are known [38] (reviewed [43]). For example, internalisation and turnover of ISG65 and ISG75 depends on ubiquitylation and sorting by the ESCRT system [7,10,8,27], but the identity of the ubiquitin ligases and deubiquitylating enzymes has remained elusive. Significant divergence within the ubiquitylation system in trypanosomes compared to animals and fungi [10] has precluded facile functional assignments based on comparative genomics of DUB and ubiquitin ligase families. Furthermore, many proteins expressed at the trypanosome surface are lineage restricted and there is little information on expression level control or turnover [6].

The gene cohort involved in suramin sensitivity is restricted to proteins that are part of the surface proteome and/or endosomal system. The cohort contains AP-1, a major player in protein targeting in many organisms. While roles for AP-1 in trafficking of p67, the major lysosomal protein of trypanosomes are known [14,22], no impact on expression or location of ISGs was observed here. The absence of canonical dileucine adaptin-binding motifs in the cytoplasmic domains of ISGs is also consistent with this finding, and suggests that AP-1 mediates an aspect of suramin-sensitivity distinct from ISG trafficking. It is possible that this is mediated *via* p67 itself, which was also identified as a suramin-sensitivity determinant and is probably directed to the lysosome *via* a route distinct to that for endocytosis of surface proteins. However, while evidence supports a role for AP-1 in p67 transport in insect stage cells, data based on proteolytic processing of p67 suggests this is not the case in bloodstream-form cells [44]. A small, but reproducible, upregulation of CatL under AP-1 knockdown was observed, suggesting that AP-1 is involved in CatL delivery to the lysosome and possibly also its maturation, but the mechanism remains unclear. These data do, however, demonstrate the presence of an ISG-independent, AP-1-dependent pathway that influences suramin sensitivity, suggesting that trafficking to the lysosome is an important factor.

**Fig 9:**
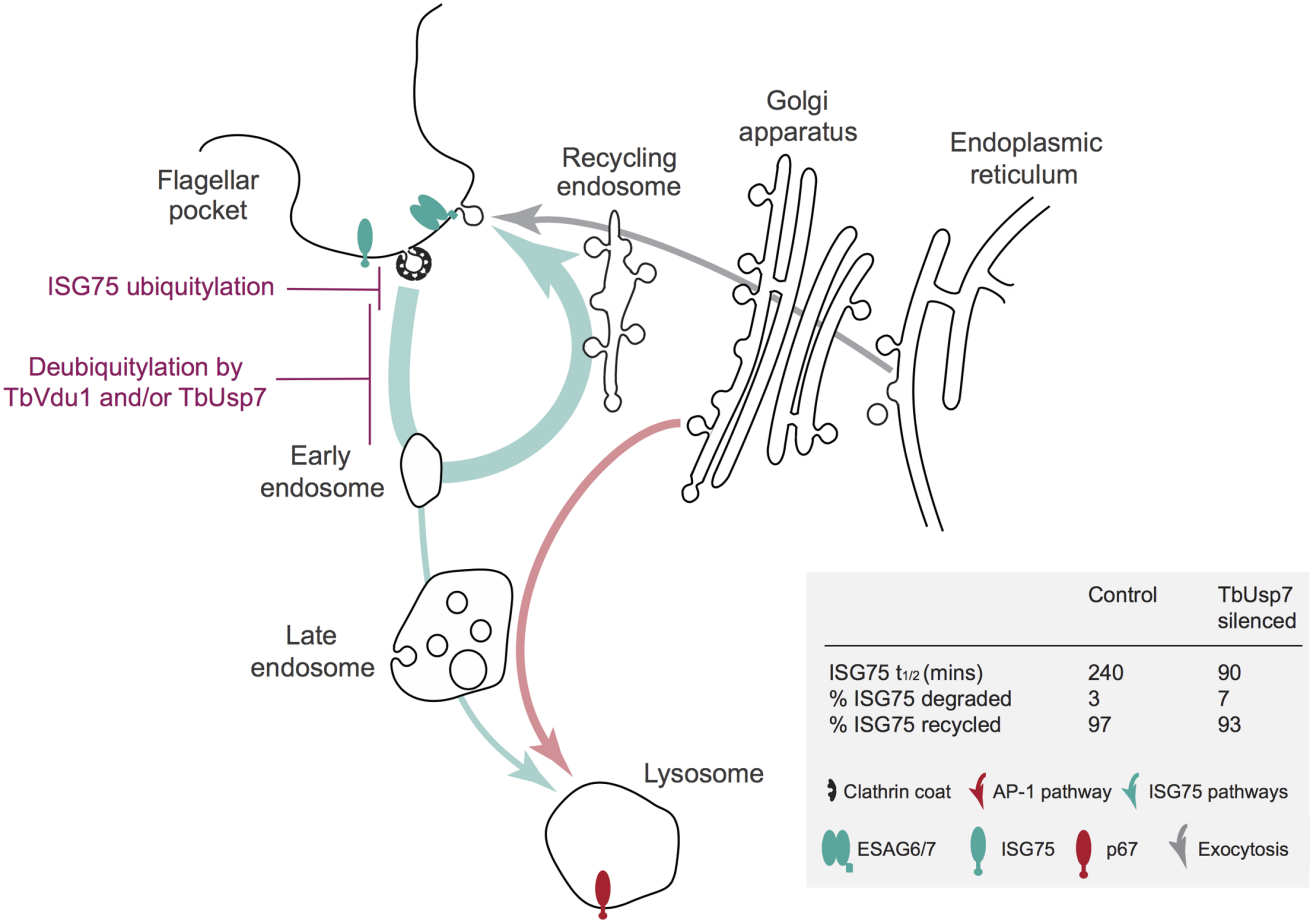
Model integrating suramin-sensitivity pathways, trafficking and ISG turnover. A simplified schematic of the trypanosome endomembrane system is shown, with the flagellar pocket at top left. Teal arrows indicate ISG degradative and recycling trafficking routes, red putative AP-1-mediated transport from the Golgi complex to the lysosome [14, 27] and gray exocytic/biosynthetic pathways. The predominant locations of ISG75, ESAG6/7 (the transferrin receptor) and p67 (the major lysosomal protein) are indicated by icons. Evidence suggests that ISG75 is ubiquitylated at, or close to the surface (magenta) and deubiquitylation by TbUsp7 and/or TbVdu1 is proposed to take place prior to the sorting step at the early endosome that selects for the recycling or degradative arm of the endocytic system. TbVdu1 is known to associate with structures in this region, whilst TbUsp7 is likely cytosolic. Approximate percent changes in the proportion of ISG75 transiting the different arms of the endocytic system are calculated from differences in half-life derived for observed ISG75 turnover changes upon TbUsp7 knockdown (Fig 5) and assuming a recycling cycle time of ~10 min. AP-1 is proposed to mediate pathways required to deliver components to the lysosome required for suramin to translocate to the cytosol.

By contrast, TbUsp7 and TbVdu1, two evolutionarily conserved DUBs, are critical to the control of ISG abundance and indicate the presence of a second, ISG-dependent pathway for suramin sensitivity. Our data suggests a model in which silencing of the respective DUB leads to increased targeting of specific ISG proteins into the degradative arm of the endocytic pathway (Fig 9). We propose that the most parsimonious interpretation is that TbUsp7 and TbVdu1 impact ISG cargo uptake by directly affecting ISG stability. This is supported by several lines of evidence; membership of the suramin-sensitivity gene cohort, demonstration of direct, and highly specific impact on ISG expression level, homology to mammalian DUBs, localisation of TbVdu1 to the endosomal region, the absence of a major impact on ISG biosynthesis and the ability to transfer knockdown sensitivity to the BiPN chimera. A direct demonstration of hyperubiquitylation has not been possible, as the expression level of the ISG75 protein itself is so greatly decreased in knockdown cells making detection of the small fraction of ubiquitylated adduct unreliable. None-the-less, all of these data support an intimate relationship between ISG turnover rates and TbUsp7 and TbVdu1.

Proteomics revealed highly specific and precise control of ISG75 and ISG65 by TbVdu1. Silencing of TbUsp7 resulted in strong downregulation of MBAP1, a membrane protein essential for both incoming and recycling membrane traffic [33], and upregulation of several ESAG products. Due to the presence of a high confidence predicted ubiquitylation site at the cytoplasmic domain of MBAP1 (Lys502, 0.96 likelihood in UbPred [45]), it is tempting to speculate that MBAP1 steady state levels are also controlled by ubiquitylation. The observed accumulation of ESAGs, including the transferrin receptor, could result from a block to endocytosis following MBAP1 downregulation. However, upregulated proteins form a relatively small cohort, while the bulk surface proteome, including for example ISG64, appear unaffected by TbUsp7 knockdown, suggesting that distinct mechanisms control expression of GPI-anchored and *trans*-membrane domain proteins. This also includes p67, the abundance of which was unaltered by the DUB knockdowns in SILAC analysis (S1 Table), suggesting very distinct mechanisms for controlling surface and lysosomal protein expression. Interestingly, a VSG-related (VR) protein, Tb927.7.180, was significantly upregulated by TbVdu1 knockdown, which provides the first evidence that these VR proteins are expressed at the surface.

TbUsp7 appears to be cytosolic [11], while TbVdu1 is, similarly to its human ortholog, membrane associated and appears to be confined to the flagellar pocket/early endocytic compartments (S1 Fig). Knockdown of neither DUB led to ISG mislocalisation, except in cells with an enlarged flagellar pocket, resulting from defective endocytosis and likely connected with decreased MBAP1 expression. Significantly, ISG65 and ISG75 demonstrate unique aspects in their trafficking and ubiquitylation, beyond differential sensitivity to TbUsp7 and TbVdu1, including increased ubiquitylation of only ISG75 at the cell surface following inhibition of endocytosis, suggesting that ISG75 is primarily ubiquitylated at the plasma membrane, while ISG65 may be modified elsewhere. Overall, these data indicate that ISG65 and ISG75 have distinct trafficking and modification pathways, as has been suggested by other studies [46,47], and which contribute to their differential impact on suramin sensitivity, despite being comparatively similar proteins. However, it is also likely that these two ISG families have distinct binding specificities, which are also relevant to accumulation of suramin. Blue-native PAGE indicates that both proteins likely exist as complexes, with ISG65 mainly present as a dimer, and ISG75 as dimers and higher order forms (S4 Fig); it is unclear how these biochemical differences connect to the distinct trafficking pathways.

In common with other eukaryotes, E3 ubiquitin ligases are likely key players in ubiquitylation in trypanosomes, a system that arose in Archaea prior to eukaryogenesis [48]. There are a large number of RING and HECT family E3s encoded in the trypanosome genome, together with representatives of the Rbx/Cullin E3 ligases. DUBs function in a temporally and spatially distinct manner to remove ubiquitin modifications, and these two systems also modulate each other by direct interactions. Stabilisation of cognate E3 ubiquitin ligases by deubiquitylation is a known aspect of DUB function [49] and DUBs are themselves ubiquitylation substrates. The mammalian ortholog of TbVdu1, is a downstream target for ubiquitylation by the Cullin-RING E3 ubiquitin ligase component of pVHL [29], a complex that acts to control expression of many proteins, including the transcription factor HIF-1α and also PAR3, which moderates endocytic pathways in mammalian cells and is associated with tumorigenesis [29]. The Rbx1 component of the equivalent complex in African trypanosomes has been targeted by RNAi and leads to defective kinetoplast replication in insect stages, but has little impact in the bloodstream form, while the relevant Cullin protein has no impact on replication in either life stage (Federico Rojas, personal communication). Significantly, a likely DUB for this complex in trypanosomes, TbVdu1 is membrane-associated and targeted to the flagellar pocket/early endosomes; this localisation is consistent with the elevated ISG75 ubiquitylation seen upon the cessation of endocytosis following clathrin RNAi knockdown. These findings support the existence of a ubiquitylation switch in trypanosomes mediating ISG75 (but not ISG65) expression levels. We have previously observed rather complex mechanisms underpinning ISG65 and ISG75 expression, such that very limited mutations within the cytoplasmic domain, altering three lysine codons to arginine, lead to significant differences in mRNA levels not reflected by changes in protein level [8]. Control of ISG75 here is very similar to that of type 2 iodothyronine deiodinase, which has a very short half-life but is stabilised by VDU1 in mammals [50].

In conclusion, we describe two pathways that contribute towards suramin sensitivity, an AP-1 dependant pathway and an ISG-dependant pathway, with a particular focus on the latter. Complex interactions between TbUsp7, ISG75 and other membrane proteins, especially MBAP1 identifies sophisticated mechanisms coordinating surface protein expression and intracellular targeting in trypanosomes. Both pathways are required for suramin-sensitivity, and we speculate that the AP-1-dependent pathway is required to maintain lysosomal conditions required for suramin-sensitivity, whilst the ISG75 pathway is needed for delivery of suramin to that compartment. The presence of a conserved Rbx/Cullin-type ubiquitylation switch, as evidenced by TbVdu1, indicates that control of ISG75 expression is likely critical to the parasite, although the normal physiological function of ISG75 remains to be determined. The essentiality of AP1 and the presence of multiple ISG75 paralogs also may explain the remarkable lack of emergence of suramin resistant strains in the wild. Finally, these data are further evidence of the high clinical value of endocytosis in the treatment of trypanosomiasis, adding suramin acquisition to a list already containing VSG recycling, antibody clearance, essentiality of many clathrin-associated proteins and N-myristoyltransferase inhibitor sensitivity [51].

## Materials and Methods

### Cell culturing of *T*. *brucei brucei*

Bloodstream form (BSF) Molteno Institute Trypanosomal antigen type (MITat) 1.2, derived from Lister strain 427 and expressing VSG221, were cultured in HMI-9 complete medium (HMI-9 supplemented with 10% heat-inactivated fetal bovine serum (FBS), 100 U/ml penicillin, 100 U/ml streptomycin and 2 mM L-glutamine) [52] at 37°C with 5% CO_2_ in a humid atmosphere, in non-adherent culture flasks with vented caps. 2T1 cells (a variant of MITat 1.2) were maintained in HMI-9 complete medium in the presence of phleomycin (1 μg/ml) and puromycin (1 μg/ml). Following transfection with stem-loop RNAi plasmids, 2T1 cells were maintained in phleomycin (1 μg/ml) and hygromycin (2.5 μg/ml) [53,54]. All TbUsp7 and TbVdu1 RNAi experiments were performed following 48 h induction with tetracycline (1 μg/ml). Cells harbouring the p2T7-AP-1γ RNAi construct were maintained in G418 (2.5 μg/ml) and phleomycin (0.5 μg/ml) as described previously [14]. Cells were maintained at densities between 10^5^ and 2.5 x 10^6^ cells/ml. Suramin EC_50_ was assessed following three days of TbVdu1 RNAi knockdown. Cells were plated at 2x10^3^ cells/ml in 96-well plates in a two-fold dilution series of suramin, starting from 1 μM. After three days growth, 20 μl resazurin (Sigma) at 125 μg/ml in PBS was added to each well and incubated for a further six hours at 37°C. Fluorescence was determined using a plate reader (Molecular Devices) with the following settings: excitation, 530 nm; emission, 585 nm; filter cut off, 570 nm. Data were processd in Excel, and non-linear regression analysis carried out in GraphPad Prism.

#### Plasmid constructs

Gene-specific RNAi fragments of 400–600 bp were amplified with PCR primers designed using RNAit [55] and cloned into pRPa^iSL^ to generate stem-loop, ‘hairpin’ dsRNA to induce RNAi knockdown [54]. The following primers were used: TbUsp7SL_F (5’-GATCGGGCCCGGTACCTTCAAGCTGTTTGGCAGTTG-3’), TbUsp7SL_R (5’-GATCTCTAGAGGATCCAACAGGGTTCGCACGATTAC-3’), TbVdu1SL_F (5’-GATCTCTAGAGGATCCAACAGGATGGGACACCTCAG-3’), TbVdu1SL_R (5’-GATCGGGCCCGGTACCACTTTGAGCAGCGCCTACAT-3’). All constructs were verified by standard sequencing methods (Source BioScience LifeSciences). TbVdu1 was *N*-terminally tagged with GFP or six myc epitopes at the native locus using pNAT^TAG^X [54]. The following primers were used to amplify a 1003 bp gene fragment: 5TbVdu1xba (5’-GATCTCTAGATTGTCATCTTCGTCGCCGGT-3’) and 3TbVdu1bam (5’-GATCGGATCCTCAGCGAAGAAAAACTCTCC-3’). Prior to introduction into trypanosomes, pRPa^iSL^ constructs and pNAT^TAG^TbVdu1 were linearised with *Asc*I or *Mfe*I, respectively, and purified by phenol:chloroform extraction.

### Quantitative real-time polymerase chain reaction (qRT-PCR)

1 x 10^8^ cells were harvested at 800 x g for 10 min at 4°C and washed with ice-cold PBS and quick-frozen in dry ice for 1 min. RNA was purified using the RNeasy mini kit (Qiagen) according to the manufacturer’s instructions. RNA concentration was quantified using an ND-1000 spectrophotometer and Nanodrop software (Nanodrop Technologies). cDNA synthesis and qRT-PCR reaction setup was performed as described previously [56]. qRT-PCR was performed using iQ-SYBRGreen Supermix on a MiniOpticon Real-Time PCR Detection System (Bio-Rad) and was quantified using Bio-Rad CFX Manager software (Bio-Rad). The following primers were used for qRT-PCR: bTub-RTF (5’-CAAGATGGCTGTCACCTTCA-3’), bTub-RTR (5’-GCCAGTGTACCAGTGCAAGA-3’); USP1 RTF (5’-GAGATGGCACCATCACTCCT-3’), USP1 RTR (5’-GTGGGCAGCACCTCTAGAAC-3’); VDU1 RTF (5’-GTCGAAAGACGTGTGGGTTT-3’), VDU1 RTR (5’-GGAGCGAGGGAAGAGAGATT-3’); ISG65-RTF (5’-GAGCATGTTGATAGAGGGATTG-3’), ISG65-RTR (5’-CATTGCTGTTCTCTGATGTCTG-3’); ISG75-RTF (5’-GAGGGCAGCGAGGCCAAG-3’), ISG75-RTR (5’-CTTCCTACGGCCCCTAATAAC-3’).

### Transfection

3 x 10^7^ bloodstream-form cells were harvested by centrifugation at 800 x g for 10 min at 4°C. Cells were resuspended in 100 ul of Amaxa Human T-cell Nucleofector solution (VPA-1002) at 4°C, mixed with 10 ug (in 5 ul) of linearised plasmid DNA and transferred to electrocuvettes. Transfection was achieved using an Amaxa Nucleofector II with Program X-001. Cells were then transferred to Tube A containing 30 ml of HMI-9 medium plus any appropriate antibiotic drug for parental cell growth. Serial dilution was performed by transferring 3 ml of cell suspension from Tube A into Tube B containing 27 ml of HMI-9 medium and repeated again by diluting 3 ml from Tube B into Tube C. 1 ml aliquots for each dilution were distributed between three 24-well plates and incubated at 37°C. After 6 h, HMI-9 containing antibiotic selection was added to the wells at the desired final concentration. Transformed cells were recovered on day 5–6 post-transfection.

### Immunofluorescence (IF)

Samples for IF were prepared as previously described (Leung *et al*., 2008). Antibodies were used at the following dilutions: mouse and rabbit anti-HA epitope IgG (both from Santa Cruz Biotechnology Inc.) at 1:1000, mouse 9E10 anti-myc (Sigma) at 1:1000, rabbit anti-GFP (Life Technologies) at 1:500, rabbit anti-ISG65 and rabbit anti-ISG75 (from P. Overath, Tubingen) at 1:1000, rabbit anti-CatL at 1:1000 (from J. Bangs, Buffalo), mouse anti-GLP-1 at 1:1000 (from D. Russell, Cornell). Secondary antibodies were used at the following dilutions: anti-rabbit Cy3 (Sigma) at 1:1000. Coverslips were mounted using Vectashield mounting medium supplemented with 4’,6-diamidino-2-phenylindole (DAPI) (Vector Laboratories, Inc.). The cells were examined on a Nikon Eclipse E600 epifluorescence microscope fitted with optically matched filter blocks and a Hamamatsu ORCA CCD camera. Digital Images were captured using Metamorph software (Universal Imaging Corp.) on a Windows XP computer (Microsoft Inc.), and the raw images were processed using Adobe Photoshop CS3 (Adobe Systems Inc.).

### Protein turnover

Protein synthesis was blocked by the addition of cycloheximide (100 μg/ml) and 1 x 10^7^ cells were harvested at various time points by centrifugation at 800 x g for 10 min at 4°C. Cells were washed in ice-cold PBS, then resuspended in 1 x SDS sample buffer and incubated at 95°C for 10 min. Samples were subjected to protein electrophoresis.

### Western immunoblotting

Whole cell lysates and hypotonic lysis fractions were prepared as previously described (Leung et al, 2008). Proteins were separated by electrophoresis on 12.5% SDS-polyacrylamide gels and then transferred to polyvinylidene difluoride (PVDF) membranes (Immobilon; Millipore) using a wet transfer tank (Hoefer Instruments). Non-specific binding was blocked with Tris-buffered saline with 0.2% Tween-20 (TBST) supplemented with 5% freeze-dried milk and proteins were detected by Western immunoblotting. The PVDF membrane was then incubated in primary antibody diluted in TBST with 1% milk for 1 h at room temperature. Antibodies were used at the following dilutions: monoclonal anti-HA (sc-7392, Santa Cruz) at 1:10,000, mouse 9E10 monoclonal anti-myc (Source Biosciences) at 1:5,000, rabbit polyclonal anti-GFP (Life Technologies) at 1:5,000, rabbit polyclonal anti-ISG65 and anti-ISG75 both at 1:10,000, KMX-1 anti-beta-tubulin at 1:2000 (Millipore), rabbit anti-CatL at 1:1000, mouse anti-GLP-1 at 1:1000. Following three washes with TBST each for 10 min, the membrane was incubated in secondary antibody diluted in TBST with 1% milk for 1 h at room temperature. Commercial secondary anti-rabbit peroxidase-conjugated IgG (A0545, Sigma) and anti-mouse peroxidase-conjugated IgG (A9044, Sigma) were used both at 1:10,000. Detection was by chemiluminescence with luminol (Sigma) on BioMaxMR film (Kodak). Densitometry quantification was achieved using ImageJ software (NIH). Blue Native PAGE followed by Western blotting experiments were carried out as described [57].

### Radioimmunoprecipitation assay (RIPA)

1 x 10^7^ cells were pelleted at 800 x g for 10 min at 4°C, washed twice in PBS and resuspended in 500 ul of Met/Cys-free RPMI-1640 medium supplemented with dialysed FBS followed by an incubation at 37°C for 1 h. Cells were pulse-labeled at 37°C for 1 h with EasyTag EXPRESS [^35^S] Protein Labeling Mix (PerkinElmer) at a specific activity of 200 uCi/ml (7 ul of 14.3 mCi/ml) and then instantly cooled on ice. Cells were pelleted at 16,000 x g for 15 sec on a table top centrifuge, washed twice with ice-cold PBS and lysed by the addition of 100 ul of RIPA buffer [25 mM Tris-HCl pH 7.5, 150 mM NaCl, 1% NP-40, 0.5% sodium deoxycholate, 0.1% SDS, and Complete Mini Protease Inhibitor Cocktail (Roche)] for 15 min on ice. Lysates were centrifuged for 30 s to remove nuclei and cell debris, and the supernatant transferred to a fresh tube. 13 μl of 10% SDS was added to the supernatant and incubated at 95°C for 5 min. Samples were diluted with 750 μl of dilution buffer (50 mM Tris-HCl pH 7.5, 1.25% Triton X-100, 190 mM NaCl, 6 mM EDTA, Complete Mini Protease Inhibitor Cocktail). 30 μl of Pansorbin (Calbiochem) (pre-washed and resuspended in dilution buffer) was added to the supernatant and incubated at 4°C for 1 h. Samples were centrifuged at 16,000 x g for 5 min and the supernatant transferred to a fresh tube. 5 μl of anti-HA antibody was added to each sample and incubated at 4°C overnight on a rotating device. Immune complexes were then isolated by the addition of 20 μl of Protein A-Sepharose on a rotating device for 1 h at room temperature. Subsequently, Sepharose beads were washed twice with wash buffer I (50 mM Tris pH 7.5, 0.1% Triton X-100, 0.02% SDS, 150 mM NaCl, 5 mM EDTA) and twice with wash buffer II (50 mM Tris pH 7.5, 0.02% Triton X-100, 1 M NaCl). A further centrifugation step was performed for 15 s at 16,000 x g and the remaining supernatant was removed by pipetting. Samples were resuspended in 1 x SDS sample buffer and denatured at 95°C for 5 min and subjected to 12.5% SDS-PAGE. Gel was then immersed in destaining solution for 20 min, washed twice with distilled water and then soaked in 1 M sodium salicylate for a further 20 min. The gel was then dried on Whatman 3MM paper for 2 h at 60°C and exposed to autoradiographic film for 1 week.

#### Densitometry

All fluorographs were scanned at 16-bit gray-scale resolution, and exposures selected to ensure the film was not saturated. In most cases the exposures shown in the figures represent over-exposed versions of the same data used in quantitation. Quantitation and background subtraction was then done with ImageJ (http://rsb.info.nih.gov/ij/).

#### Concanavalin A (ConA) uptake assay

1.5 x 10^6^ cells were harvested at 800 x *g* for 10 min at 4°C, washed with serum-free HMI-9 supplemented with 1% bovine serum albumin (BSA) and incubated in serum-free HMI-9 with 1% BSA for 20 min at 4°C. Cells were then incubated with 50 μg/ml of fluorescein isothiocyanate (FITC)-conjugated ConA (Vector Labs FL-1001) for 20 min at 4°C. Chase was initiated by incubating cells at 37°C with samples harvested at various time points by immediately placing on ice and washing with vPBS at 800 x *g* for 10 min at 4°C. Labelled cells were fixed and co-stained as described for IF.

### Stable isotype labeling by amino acids in cell culture (SILAC) labeling

HMI11 for SILAC was prepared essentially as described in [58]: IMDM depleted of L-Arginine, L-Lysine (Thermo) and 10% dialysed (10 kDa molecular weight cutoff) fetal bovine serum (Dundee Cell Products) was supplemented with 4 ug/ml folic acid, 110 μg/ml pyruvic acid, 39 μg/ml thymidine, 2.8 μg/ml bathocuproinedisulfonic acid, 182 μg/ml L-cysteine, 13.6 μg/ml hypoxanthine, 200 μM β-mercaptoethanol, 0.5 μg/ml Phleomycin and 2.5 μg/ml Hygromycin. Finally either normal L-Arginine and L-Lysine (HMI11-R0K0), or L-Arginine U−^13^C_6_ and L-Lysine 4,4,5,5-^2^H_4_ (HMI11-R6K4) (Cambridge Isotope Laboratories) were added at 120 uM and 240 uM respectively. RNAi was induced by addition of 1 μg/ml tetracycline. At the indicated times, equal numbers of induced and uninduced cells, grown in the presence of HMI11-R0K0 or HMI11-R6K4 respectively, were mixed, harvested by centrifugation, washed twice with PBS containing Complete Mini Protease Inhibitor Cocktail (Roche) then resuspended in Laemmli buffer containing 1 mM dithiothreitol and stored at -80°C. TbUsp7 and TbVdu1 RNAi samples were generated in duplicate and triplicate, respectively, with each replicate representing a different clone. One label swap was performed in each set of replicates. The heavy isotope incorporation at steady state was determined from one gel slice (60–80 kDa) of a control experiment omitting induction. Samples were sonicated and aliquots containing 5 x 10^6^ cells were separated on a NuPAGE bis-tris 4–12% gradient polyacrylamide gel (Invitrogen) under reducing conditions. The sample lane was divided into eight slices that were excised from the Coomassie stained gel, destained, then subjected to tryptic digest and reductive alkylation. Liquid chromatography tandem mass spectrometry (LC-MS/MS) was performed by the Proteomic Facility at the University of Dundee. The eight fractions obtained from SDS-PAGE were subjected to LC-MS/MS on a UltiMate 3000 RSLCnano System (Thermo Scientific) coupled to a LTQ OrbiTrap Velos Pro (Thermo Scientific) and mass spectra analysed using MaxQuant version 1.5 [59] searching the *T*. *brucei brucei* 927 annotated protein database (release 8.1) from TriTrypDB [60]. Minimum peptide length was set at six amino acids, isoleucine and leucine were considered indistinguishable and false discovery rates (FDR) of 0.01 were calculated at the levels of peptides, proteins and modification sites based on the number of hits against the reversed sequence database. SILAC ratios were calculated using only peptides that could be uniquely mapped to a given protein. If the identified peptide sequence set of one protein contained the peptide set of another protein, these two proteins were assigned to the same protein group.

## Supporting Information

S1 Fig TbVdu1 is membrane-associated and has a subcellular localisation consistent with an early endocytic compartment. (A) ^6myc^Vdu1 is found in the membrane fraction following hypotonic lysis. S, soluble; W, wash; P, pellet. (B) Western blot shows specific expression of ^GFP^Vdu1. Coomassie stained gels show loading. (C) ^GFP^Vdu1 localises to a region proximal to the kinetoplast. The dashed regions are presented in the right hand panels. Cells were co-stained with DAPI to visualise the nuclear and kinetoplast DNA. Scale bar = 2 μm. (D) TbVdu1 knockdown has no effect on cell proliferation. Cells were grown over a period of 5 days in the presence (white squares) or absence (black squares) of TbVdu1 RNAi and cell numbers monitored. (E) TbVdu1 RNAi and EC_50_ analysis confirms the contribution of TbVdu1 to suramin efficacy. TbVdu1 RNAi strain was grown +/-tetracycline and then subjected to EC_50_ analysis; non-linear regression analysis was carried out in Graphpad Prism. Representative plot is shown; error bars represent standard error from a quadruplicate assay. Relative change in EC_50_ following RNAi in three independent cell lines is shown in the box.
(PSD)

S2 Fig ConA uptake assay in the presence and absence of TbUsp7 or TbVdu1 RNAi. Following induction of RNAi, cells were incubated with FITC-conjugated ConA and uptake was monitored at 0, 10, 20, 30 and 60 min. Note that only BigEye cells are presented for TbUsp7 induced for RNAi. Induced cells that do not display the BigEye phenotype have normal ConA uptake as in uninduced cells. All cells were co-stained with DAPI to visualise nuclear and kinetoplast DNA. Scale bar = 2.5 μm.
(PSD)

S3 Fig TbUsp7 knockdown does not impact on clathrin steady state levels. TbUsp7 RNAi was induced for 48 h and levels of clathrin were monitored by Western blotting using anti-clathrin heavy chain (CLH) antibody.
(PSD)

S4 Fig ISG65 and ISG75 are multimeric. Bloodstream-form cell lysates were subjected to Blue Native PAGE, followed by western blotting. Molecular weight standard masses are given in kDa on the left hand side.
(PSD)

S1 Table Raw SILAC proteomics data. Protein groups output table of the MaxQuant analysis of TbUsp7 RNAi (26h and 48h) and TbVdu1 RNAi (48h). False identifications (contaminants, reverses) and peptide IDs were removed from the list. Columns G-K were added and list the normalised ratios (induced/uninduced) for each independent experimental replicate (grey), the respective average (orange) and standard deviation (light orange). ND; not detected.
(XLSX)
