# Correction: Modulation of the Surface Proteome through Multiple Ubiquitylation Pathways in African Trypanosomes

**DOI:** 10.1371/journal.ppat.1005291

**Published:** 2015-11-23

**Authors:** Martin Zoltner, Ka Fai Leung, Sam Alsford, David Horn, Mark C. Field

There are typographical errors in Table 1. Some of the sub-rows were separated into individual rows, when they should be included in prior rows. Please see the corrected table here.

**Table 1:** Percentage abundance of selected protein groups upon TbUsp7 and TbVdu1 RNAi derived from normalised SILAC ratios.

Annotation	Protein group	Protein abundance upon RNAi (percent relative to non-induced)			Predicted features	Predicted *Trans-*membrane domain	PredictedN-terminal signal	Number of lysines in cytoplasmic domain	Predicted GPI-anchor	Sequence length(inclusive signal seq.)
		TbUsp7 26h	TbUsp7 48h	TbVdu1 48h						
USP7	Tb927.9.14470	28 *	13 *	101 (+/- 3)	USP7_C2 superfamily	No	No	NA	NA	1161
VDU1	Tb927.11.12240	ND	105 *	ND	*Peptidase_C19 superfamily*	No	No	NA	NA	790
ISG75	Tb927.5.390	43 (+/- 4)	40 (+/- 34)	52 (+/- 5)	ISG65-75 superfamily	468–490	Yes	5	NA	522
	Tb927.5.400					468–490		4		522
	Tb927.5.350					468–490		4		522
	Tb927.5.360	42 (+/- 3)	39 (+/- 26)	53 (+/- 4)	ISG65-75 superfamily	469–491	Yes	5	NA	523
	Tb927.5.370	ND	ND	64 (+/- 23) *	ISG65-75 superfamily	469–491	Yes	5	NA	523
ISG65	Tb927.2.3280	96 (+/- 2)	117 (+/-5)	69 (+/- 8)	ISG65-75 superfamily	386–408	Yes	4	NA	436
	Tb927.2.3290					388–410		4		436
	Tb927.2.3300					388–410		4		436
	Tb927.2.3310					388–410		3		436
	Tb927.2.3320	97 (+/- 2)	109 (+/- 41)	45 (+/- 2)	ISG65-75 superfamily	387–409	Yes	3	NA	437
	Tb927.2.3270	90 (+/- 2)	105 (+/- 22)	62 (+/- 8)	ISG65-75 superfamily	388–410	Yes	4	NA	436
	Tb11.v5.0231					469–491			5	523
	Tb11.v5.0731					388–410			2	430
ISG-related	Tb927.5.630	75 (+/- 3)	79 (+/- 16)	76 (+/- 11)	ISG65-75 superfamily	349–372	Yes	4	NA	401
ISG64	Tb927.5.1390*	92 (+/- 3)	98 (+/- 2)	87 (+/- 19)	ISG65-75 superfamily	376–398	Yes	4	NA	434
	Tb927.5.1410					377–399		4		435
	Tb927.5.1430	97 (+/- 9)	107 (+/- 5)	94 (+/- 20)	ISG65-75 superfamily	376–398	Yes	4	NA	434
MBAP1	Tb927.11.13130	50 (+/- 1)	29 (+/- 2)	101 (+/- 11)	acidic phosphatase [33]	459–481	Yes	2	No	524
putative type I membrane protein 1	Tb927.7.470	38 (+/- 1)	32 *	85 (+/- 4)		182–204	Yes	4	No	297
putative type I membrane protein 2	Tb927.9.11480	45 (+/- 4)	34 (+/- 3)	87 (+/- 2)		512–537	Yes	1	No	561
putative type IV membrane protein	Tb927.11.7550	51 (+/- 2)	38 *	97 (+/- 7)*		49–71,112–134,141–163,190–212	No	3	No	221
VAMP7B	Tb927.5.3560	70 (+/- 3)	62 (+/- 12)	81 (+/- 21)	Vesicle-associated membrane protein	184–206	No	No	No	796
TPR-repeat protein	Tb927.11.810	46 *	55 (+/- 4)	97 (+/- 4)	Tetratrico peptide repeat [42]	No	No	No	No	216
ESAG5	Tb11.v5.0826	142 (+/- 2)	170 (+/- 26)	132 (+/- 12)	potential lipid or lipo-polysaccha ride binding [36,37]	No	Yes	NA	No	464
	Tb927.7.6860									480
ESAG6	Tb927.7.3250	132 (+/- 17)	202 (+/-56)	101 (+/- 43)*	Transferrin receptor	No	Yes	NA	Yes	397
ESAG7	Tb927.7.3260					No	Yes	NA	No	339
ESAG2	Tb927.11.14620	114 (+/- 8)	115 (+/- 21)	85 (+/- 3)		No	Yes	NA	Yes	458
VSG-related protein	Tb927.7.180	126 (+/- 17)	139 *	117 (+/- 4)*	VSG-related	No	Yes	NA	No	437

Values represent average percentage protein abundance relative to uninduced cells ± standard deviation. TbUsp7 and TbVdu1 RNAi samples were analysed at indicated time points in experimental duplicate and triplicate, respectively. Asterisks mark proteins not quantified in all replicates. Polypeptide features predicted using TMHMM2 for *trans-*membrane domains [39], signalP for N-terminal ER-targeting signal [40], PredGPI for GPI-anchor addition C-terminal signal sequence [41] and TPRpred for tetratrico peptide repeats [42]. Predictions are given as Yes, No responses or sequence positions (inclusive signal sequence) using default parameters. In most cases the topology of the protein is experimentally known or predictable based on close homology of experimentally derived information. All protein hits shown have been inspected for their genomic context and integrity. Protein groups consist of indistinguishable paralogs, sharing identical quantified peptides. For the complete quantification data see S1 Table. NA; not applicable, ND; not detected.
