# Correction: Modulation of the Surface Proteome through Multiple Ubiquitylation Pathways in African Trypanosomes

**DOI:** 10.1371/journal.ppat.1005432

**Published:** 2016-02-01

**Authors:** The PLOS Pathogens Staff

## Notice of Republication

S1 Table was published in error. The error was corrected in the HTML version of this article on January 8, 2016, and the corrected table is available in the supporting information of this article.

## Supporting Information

S1 File Corrected S1 Table. (XLSX)
